# Mechanism of lncRNA-ANRIL/miR-181b in autophagy of cardiomyocytes in mice with uremia by targeting ATG5

**DOI:** 10.1371/journal.pone.0256734

**Published:** 2021-09-01

**Authors:** Ying Xu, Jing Chen, Minmin Wang, Rizhen Yu, Wenly Zou, Wei Shen

**Affiliations:** 1 Department of Urology, Zhejiang Provincial People’s Hospital, People’s Hospital of Hangzhou Medical College, Zhejiang, China; 2 Department of Urology, Tongde Hospital of Zhejiang Province, Zhejiang, China; 3 Department of Nephrology, Zhejiang Provincial People’s Hospital, People’s Hospital of Hangzhou Medical College, Zhejiang, China; The Ohio State University College of Medicine, UNITED STATES

## Abstract

**Objectives:**

This study is to investigate whether the cardiac microvascular endothelial cells (CMECs) can regulate the autophagy of cardiomyocytes (CMs) by secreting lncRNA-ANRIL/miR-181b exosomes, thus participating in the occurrence of uremic cardiovascular disease (CVD).

**Methods:**

A 5/6 nephrectomy uremia model was established, with the mice injected with ANRIL-shRNA lentivirus vector, miR-181b agomir, and related control reagents, containing the serum creatinine and urea nitrogen measured. The renal tissue sections of mice were stained with Periodic Acid-Schiff (PAS), TUNEL, and Hematoxylin-Eosin (HE) performed on myocardial tissue sections of mice. ANRIL-shRNA, miR-181b mimics, and related control reagents were transfected into CMECs, in which the exosomes were extracted and co-cultured with CMs. The expressions of ANRIL, miR-181b and ATG5 were detected by qRT-PCR, and the expressions of autophagy related proteins by Western blot, as well as the binding of ANRIL and miR-181b by the double luciferase reporter gene experiment.

**Results:**

ANRIL down-regulation or miR-181b up-regulation can increase the weight of mice with uremia, as well as the expressions of p62 and miR-181b, and reduce the content of serum creatinine and urea nitrogen, the damage of kidney and myocardial tissues, the number of apoptotic cells in myocardial tissues, as well as the expressions of ANRIL, ATG5, Beclin1, and LC3. CMs can absorb the exosomes of CMECs. Compared with IS+ CMEC-Exo group, the expressions of ANRIL and ATG5 in CMs of IS+ CMEC-Exo + sh lncRNA ANRIL and IS+CMEC-Exo+miR-181b mimics groups was down-regulated, as well as the expressions of ATG5, Beclin1, and LC3, while miR-181b expression was up-regulated as well as P62 expression.

**Conclusions:**

CMECs can regulate autophagy of CMs by releasing exosomes containing ANRIL and miR-181b.

## Introduction

Chronic kidney disease (CKD) has become a global public health problem with high morbidity and mortality, among which cardiovascular disease (CVD) is the main cause of death [1]. Therefore, it is necessary to clarify the pathogenesis and interventions of CVD in uremia. As an essential regulatory RNA molecule, lncRNAs have been reported to be involved in the occurrence of renal inflammation in kidney-related diseases [2], which are becoming a potential key regulator in various CVDs at the same time. However, it is still unclear whether lncRNAs are involved in the pathogenesis of uremic cardiomyopathy, with the in-depth molecular regulatory mechanism to be explored. Therefore, it is of great significance to explore the specific mechanism of cardiovascular damage in uremia from the molecular or cellular level to find effective interventions.

It has been reported that the communication between cardiac microvascular endothelial cells (CMECs) and cardiomyocytes (CMs) plays an important role in the pathogenesis of cardiomyopathy, during this process, exosomes are important mediators. In diabetic cardiomyopathy (DCM), exosomes derived from CMECs can be absorbed by CMs, to increase the content of Mst1 protein in CMs, inhibiting the autophagy of high glucose (HG) cells (25mmol/l) and promoting the apoptosis [3]. More and more studies have shown that exosomes containing secreting factors of CMECs can effectively regulate cardiac functions and positively or negatively affect cardiac remodeling [4], suggesting that in uremia cardiomyopathy, lncRNAs encapsulated in exosomes may regulate the autophagy and apoptosis of CMs through CMECs and CM corsstalk, thus regulating the occurrence and development of the disease.

ANRIL is a kind of antisense non-coding RNA at the cell cycle kinase inhibitor 4B (INK4B), which is associated with epigenetic silencing of INK4A with its expression. INK4b-ARF-INK4a plays an important role in regulating of cell cycle, senescence and apoptosis [5], which has been proved that ANRIL expression can be up-regulated in many human tumors [5, 6]. The inhibition of ANRIL expression can inhibit the proliferation, invasion, and migration of many cancer cells and promote the apoptosis of cancer. In the study of Cho et al., ANRIL expression in peripheral blood of patients with coronary heart disease (CHD) was significantly different from that of healthy people. The imbalance of ANRIL could lead to abnormal proliferation of CMs and coronary artery disease (CAD) [7]. Similarly, Cai et al. proved that ANRIL highly expressed in podocytes of diabetic nephropathy [8]. And according to the study by Dai et al., ANRIL was up-regulated in myocardial tissue of mice with diabetes mellitus. However, its mechanism remains to be further elucidated [9].

Rapid advances in bioinformatics indicate that lncRNAs may potentially interact with miRNAs to modulate their regulatory roles. In the previous studies, our research group predicted with biological methods and online database TargetScan (http://www.targetscan.org/), and found that there are possible regulatory targets between ANRIL and miR-181b. Therefore, this study aims to investigate the regulatory effects of ANRIL / miR-181b on the autophagy in mice with uremia.

## Materials and methods

### Construction of a 5/6 nephrectomy uremia model

This study was carried out in accordance with the Guide for the Care and Use of Laboratory Animals issued by the Institute of Laboratory Animal Resources of the Life Science Committee of the National Research Council, with the experimental protocol approved by the Ethics Committee of Zhejiang Provincial People’s Hospital (permit number: 2019–044). C57BL/6J mice were purchased from GemPharmatech Co.,Ltd. (Jiangsu, China), including male mice aged ten weeks, with six mice in each group, which were injected intraperitoneally with 10% pentobarbital sodium solution (150 μl), fixed in supine position on the operating table, and disinfected with 0.5% chlorhexidine solution in their abdominal. The left kidney of mice was exposed through the left abdominal incision, and adrenal gland and perirenal fat were separated from the kidney to dissociate the left kidney. After ligating a polyglycolic acid suture line in the left kidney (about 1/3 of each kidney volume), the upper and lower poles were cut off by ophthalmic scissors the hemostasis was compressed with the gelatin sponge for 2 min. After resection, the remnant kidney was reset to the renal fossa, covering the organs and peritoneum, and the peritoneum and skin were sutured layer by layer. One week after surgery, the right kidney was exposed in the same manner. The arteriovenous and ureter of the right kidney were ligated and the right kidney was removed.

### Measurement of weight, serology and urine indexes

D0 was counted from the beginning of preparation for the experiment. On D3, 2/3 of left kidney was resected, and 5/6 of right kidney was resected on D9, with the weight measured and recorded time every three days, up to D48. Blood samples were collected from the tail vein of mice on D0 and D48 (39 days after 5/6 nephrectomy), respectively. Serum creatinine was measured to evaluate the degree of kidney injury. The mice were placed in a metabolic cage 24h before death to collect, with urine volume collected, and urine protein detected by coomassie brilliant blue.

### Hematoxylin-Eosin (HE) and Periodic Acid-Schiff (PAS) staining

Mice in each group were sacrificed, to collect myocardial and kidney tissues. The collected tissues were fixed with 4% formalin in PBS and then embedded with paraffin. The embedded tissues were sectioned into 5 μm, and then performed with HE staining and PAS staining according to the method described previously [10]. Samples on the sections were analyzed under the light microscopy.

### Construction, packaging, and concentration of lncRNA-ANRIL lentiviral vectors

Upstream and downstream specific amplification primers were designed, and restriction sites were introduced to synthesize ANRIL-shRNA, which was loaded into a lentivirus plasmid vector. Lentivirus shuttle plasmids and the original plasmids of their auxiliary packaging were prepared, which were all extracted with high purity and endotoxin-free, respectively, and co-transfected into HEK293T cells. Complete medium was replaced 6h after transfection. After culturing for 48h, the cell supernatant rich in lentivirus particles was collected respectively, and concentrated by super centrifugation.

### Construction and grouping of uremia mice model with down-regulated ANRIL and up-regulated miR-181b expression

Thirty-six C57BL/6J mice were randomly divided into 6 groups, including Sham group, Model group, Model+sh NC group, Model+sh lncRNA ANRIL group, Model+NC agomir group, and Model+miR-181b agomir group, with 6 rats in each group. Mice in Model + sh NC, Model + sh lncRNA ANRIL, Model + NC agomir and Model + miR-181b agomir groups were injected with NC shRNA, ANRIL-shRNA, NC agomir and miR-181b agomir via coronary artery, respectively.

### TUENL staining

According to the manufacturer’s recommendations, the apoptosis was detected with the TUNEL detection kit (Roche, Penzberg, Germany) in myocardial and kidney tissues, which were fixed with 4% paraformaldehyde in PBS at 37°C for 1h, and permeabilized with 0.1% Triton X-100 in 0.1% sodium citrate for 2 min. After washing in PBS, sections were incubated with the TUNEL reaction mixture at 37°C for 1h, and then the stained cells were observed with fluorescence microscopy (Eclipse TE300, Nikon, Japan).

### Isolation and culture of CMECs and CMs

C57BL / 6J mice were sacrificed, to remove the hearts quickly under aseptic conditions, with the blood washed with PBS solution, as well as valves, large vessels, and connective tissue removed and soaked in 75% alcohol for 30s to inactivate the endothelial cells in the epicardium. After washing with PBS solution, the myocardial tissues were cut, with 4 ml of 0.2 g/L type II collagenase added, and digested in a 37°C water bath for 5 min, then 4 ml of 0.25 g/L trypsin was added, gently blew and mixed, and digested in a 37°C water bath for 5–10 min. After 200 mesh screening, DMEM medium containing 100 mL /L fetal bovine serum (FBS) was added and centrifuged (1000 r/min, 10 min). After discarding the supernatant, the cells were resuspended in DMEM containing 150 ml/L FBS, with the cell density of 10^5^/ml, then inoculated into the culture bottle and cultured in a constant temperature incubator at 37°C with 5% CO_2_ for 6 h, with the non-adherent cell removed. After that, the solution was changed every 3–4 days, and CMECs were subcultured when they grew into the monolayer. All the cells used in the experiment grew steadily between the second and third generations.

The ventricular muscle was cut to 1 mm for each under aseptic conditions, with 0.125% trypsin added, and then digested in water bath at 37°C for 5–6 min, to collect the digestive juice into DF12 containing 10% FBS, which was digested repeatedly for 6–8 times. After filtration, the digestive liquid was centrifuged at 1500r/min for 7 min, and then the cells were resuspended with DF12 medium, with the cell density of 10^5^/ml. Subsequently, the cells were incubated in a constant temperature incubator at 37°C with 5% CO2 for 2 h. The cell suspension was aspirated and cultured in DF12 containing 10% FBS. When CMs grew into the monolayer, the cells were passaged. All the cells used in the experiment grew steadily between the second and third generations.

### Cell proliferation assay

CMECs were treated with complete medium at the concentration of 0 μmol/L, 100 μmol/L, 250 μmol/L, 500μmol/L, 1000 μmol/L IS for 48h, with 20 μl CCK-8 solution added to each mesh of 96-mesh plates, incubated at 37°C with 5% CO_2_, and saturated humidity. After 4h of incubation, the absorbance was measured at 450 nm with a microplate reader (Thermo Fisher Scientific).

### Cell transfection

ANRIL-shRNA was knocked down ANRIL in CMECs. Nontarget shRNA lentiviruses (NC-shRNA) were used as the negative control. miR-NC and miR-181b mimics were obtained from GeneChem Company (China). Transfections were conducted with Lipofectamine 2000 (Invitrogen, USA) based on the manufacturer’s protocol.

### Extraction and identification of exosomes from CMECs

CMECs were cultured in DMEM containing 10% FBS at 37°C with 5% CO2 for 48h. When the cell density reached 1×10^6^ ~ 1×10^7^/ml, the cell culture supernatant was collected. The exosomes were extracted by SBI kit (system biosciences, USA) according to the manufacturer’s recommendations, their morphology was observed and photographed under electron microscope, and their size was was analyzed by particle size instrument. The expressions of HSP70, CD63, and TSG101 were detected by Western blot.

### Fluorescence confocal experiment

The uptake of exosomes by CMs was detected by fluorescence confocal assay, with PKH67 stain added to the exosome solution and thoroughly mixed and incubated for 10 min. The labeled exosomes and CMs with a density of 1 × 10^5^/ml were co-cultured into a six-mesh plate containing a cover glass for some time, which was gently cleaned with PBS and fixed with 4% paraformaldehyde solution at room temperature for 15 min. After washing with PBS for 3 times, the nuclei were stained with DAPI, with the anti-fluorescence quenching agent added, to cover the slide, and the images were taken under the confocal laser scanning microscope.

### Quantitative real-time PCR (qRT-PCR)

The expressions of lncRNA-ANRIL, miR-181b, and ATG5 in mouse tissues and cells were detected, to extract the total RNA through TRIzol® Reagent (CA, USA). cDNAs were synthesized from 0.5 μg mRNA by a cDNA synthesis kit (Applied Biosystems, Japan). qRT-PCR was performed with an ABI Prism 7500 instrument (Applied Biosystems, Japan), and then the cells were incubated at 37°C for 15 min, followed by 95°C for 3 min, 95°C for 12 s, and 62°C for 40 s, for 40 cycles. Each sample was treated with 3 replicates for 3 times. The expression fold change was calculated according to the comparative Ct (ΔΔCt) method. All primers are presented in Table 1.

**Table 1:** Primer sequences.

Genes	Primers (3’-5’)
lncRNA-ANRIL	F: TTATGCTTTGCAGCACACTGG
	R: GTTCTGCCACAGCTTTGATCT
miR-181b	F: ACACTCCAGCTGGGAACATTCATTGCTGTCGG
	R: TGGTGTCGTGGAGTCG
ATG5	F: GAATATGAAGGCACACCCCTGAAATG
	R: GTACTGCA TAATGGTTTAACTCTTGC
GAPDH	F:CCTCAAGATTGTCAGCAAT
	R:CCATCCACAGTCTTCTGAGT

### Western blot assay

The proteins expression of HSP70, CD63, TSG101, ATG5, Beclin1, p62, and LC3II/I were detected. And quantitative analysis was conducted with BCA protein quantitative kit (Thermo Fisher Scientific, USA) before the total proteins were extracted, which were transferred to PVDF membrane (Millipore, USA) after sodium salt-Polyacrylamide gel electrophoresis (SDS-PAGE). At room temperature, the membrane was blocked with a TBS Tween‐20 buffer containing 5% bovine serum albumin (BSA) for 1h. The antibodies used at manufacturer-recommended dilutions were purchased from Cell Signaling Technology. After washing, the blot was incubated with HRP-conjugated secondary antibody (Santa Cruz, USA) for 1h. The protein bands were developed with electro‐chemiluminescence (ECL) reagents (Millipore, Billerica, MA, USA), and images were acquired with the ChemiDoc Imaging system. Western blot assay was repeated at least three times.

### Luciferase reporter assay

lncRNA-ANRIL fragment containing predicted miR-181b binding site was amplified by PCR and inserted into pGL3 alkaline luciferase vector. Site-directed mutagenesis was carried out with a quickchange XL site directed mutagenesis kit. The cells inoculated in 24 mesh plates were co transfected with luciferase reporter molecule and miR-181b mimics or NC with Lipofectamine 2000. After 48 h, luciferase activity was measured with a dual luciferase reporter gene assay system (Promega).

### Statistical analysis

All statistical analyses were performed with SPSS 20.0 software. Data were expressed as mean ± SD. Results were analyzed by one-way ANOVA with S-N-K multiple comparison test. *p* < 0.05 showed significant difference.

## Results

### Construction of uremic mouse model and expression of ANRIL/miR-181b in the myocardium

**Fig 1:**
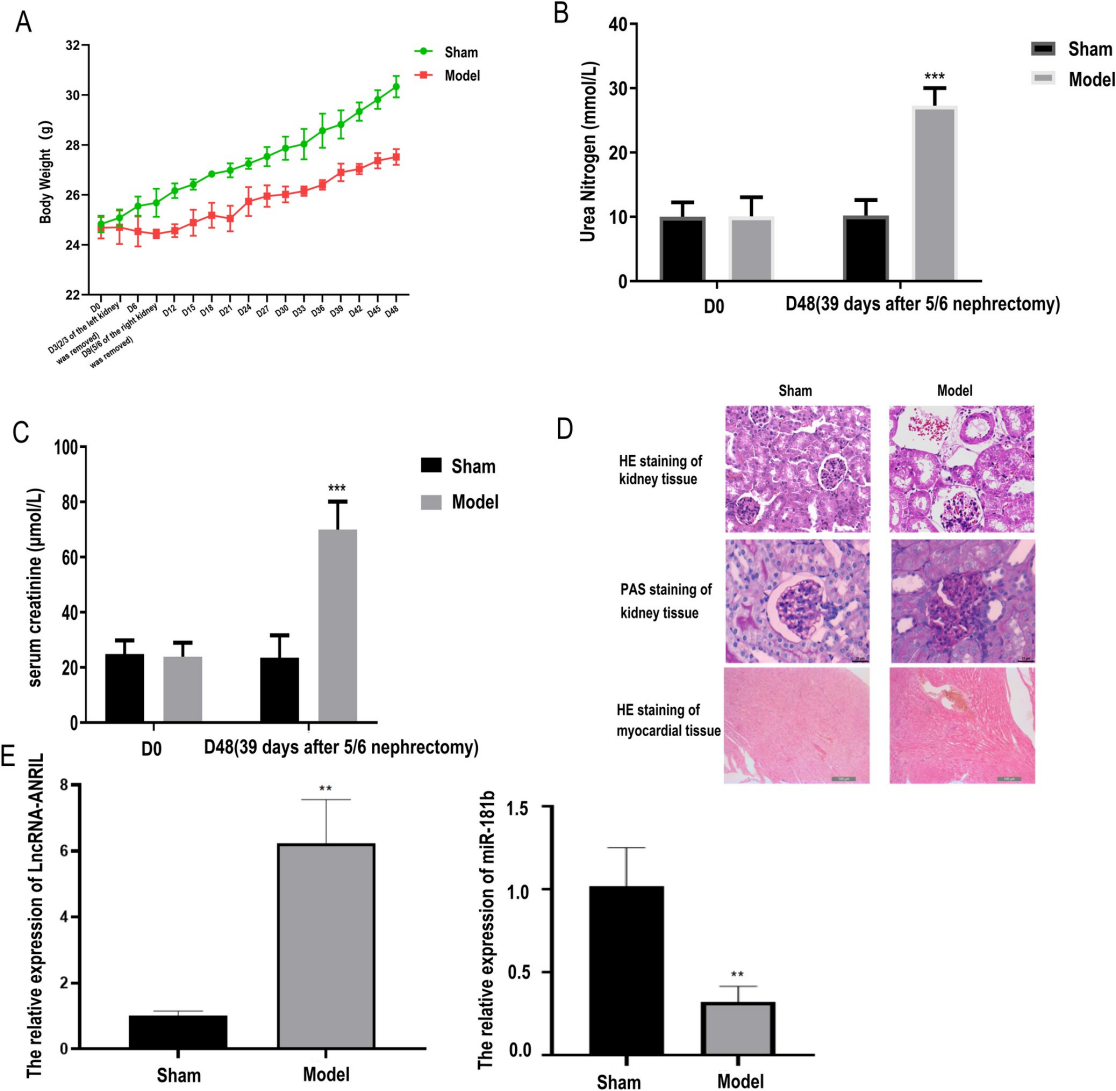
Validation of uremic mouse model and expression of lncRNA-ANRIL/miR-181b in the myocardium. (A) Changing trend of mouse body weight on D0-48. From D0, the body weight was measured and recorded at the same time every 3 days until D48. (B) Changes of urea nitrogen in mice on D0 and D48. (C) Changes of serum creatinine in mice on D0 and D48. (D) Histopathological changes in the kidney tissue of mice detected by HE and PAS staining. Histopathological changes in the myocardial tissue of mice detected by HE staining detected. (E) lncRNA-ANRIL/miR-181b expression in mice myocardium detected by qRT-PCR. ** *P*<0.01 vs. Sham group, *** *P*<0.001 vs. Sham group.

Compared with the Sham group, mice’s weight in the Model group increased slowly after operation, indicating that nephrectomy can reduce the rising trend of mice weight (Fig 1A). On D0, no significant difference in urine nitrogen and serum creatinine content between the two groups was found, but the urinary nitrogen and serum creatinine content in the Model group was significantly higher than that in the Sham group on D48 (*P* < 0.001) (Fig 1B and 1C). The results of HE staining and PAS staining showed that the renal glomeruli of the Sham group was normal without any damage. And the glomerulus structure was destroyed, with the renal tubules atrophied, secretions appeared in the lumen, mesangial cells proliferated, and the mesangial matrix increased in the Model group, indicating that the kidney of the Model group was seriously damaged. According to HE staining, the Sham group’s myocardial tissue was intact, and the cell morphology was normal, with no damage. In the Model group, myocardial tissue was damaged seriously, cell atrophy and deformation, local cell death (Fig 1D). Compared with the Sham group, ANRIL expression significantly increased in the Model group, while miR-181b expression significantly decreased in the Model group (Fig 1E).

### ANRIL down-regulation or miR-181b up-regulation can regulate serum and urine indexes, histopathological changes, and apoptosis of kidney and myocardium in mice

**Fig 2:**
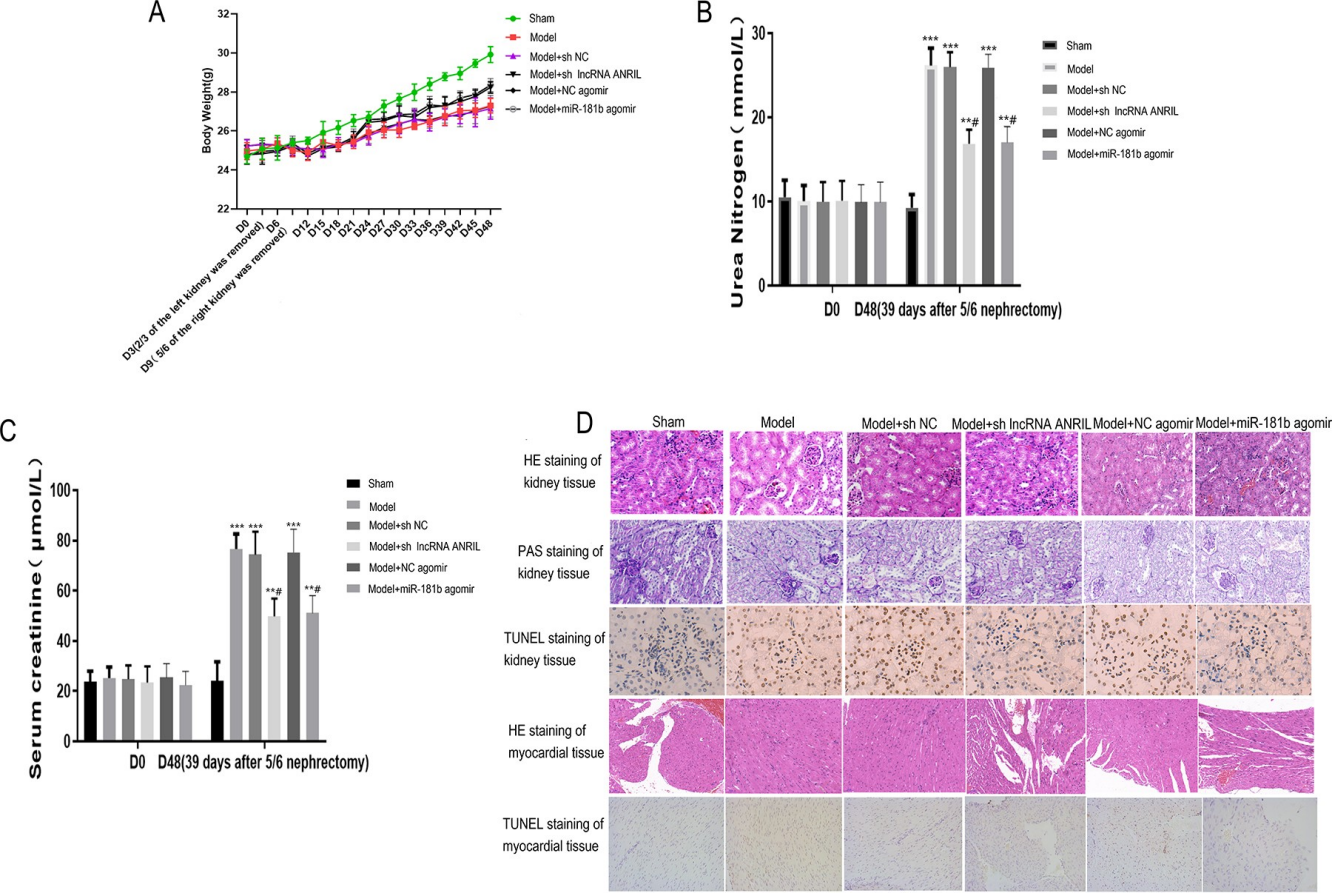
Effects of lncRNA-ANRIL down-regulation or miR-181b up-regulation on serum and urine indexes, histopathological changes and apoptosis of kidney and myocardial cells in mice. (A) Change trend of mice body weight on D0-48. From D0, the body weight was measured and recorded at the same time every 3 days until D48. (B) Changes of urea nitrogen in mice on D0 and D48. (C) Changes of serum creatinine in mice on D0 and D48. (D) Histopathological changes in the kidney tissues of mice detected by HE and PAS staining. Histopathological changes in the myocardial tissues of mice detected by HE staining. The apoptosis of kidney and myocardium tissues detected by TUNEL staining. ** *P*<0.01 vs. Sham group, *** *P*<0.001 vs. Sham group, # *P*<0.05 vs Model group.

**Fig 3:**
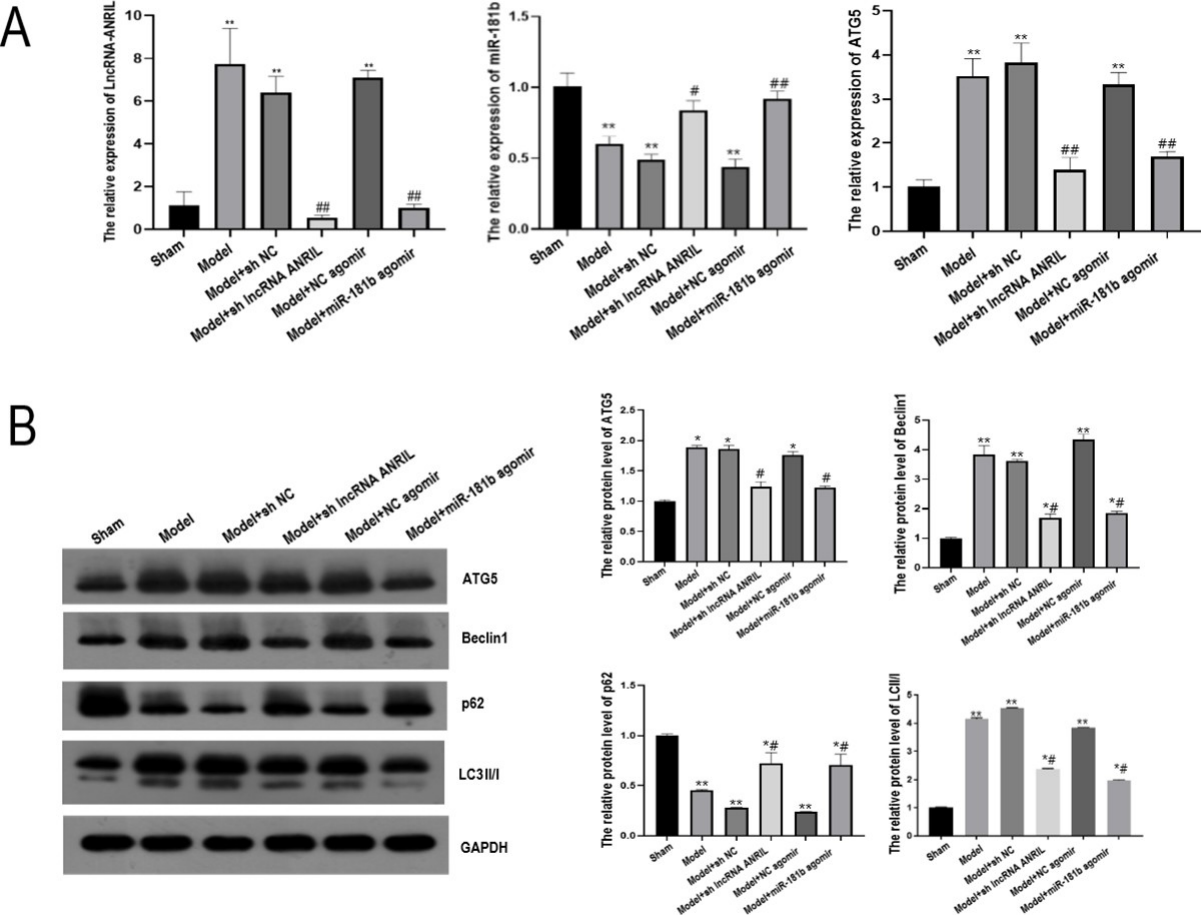
Effects of lncRNA-ANRIL down-regulation or miR-181b up-regulation on the expressions of lncRNA-ANRIL, ATG5, miR-181b and autophagy-related proteins in mice myocardium. (A) Expressions of lncRNA-ANRIL, ATG5, and miR-181b detected by qRT-PCR. (B) Protein expressions of ATG5, Beclin1, p62, and LC3II/I detected by Western blot. * *P*<0.05 vs. Sham group, ** *P*<0.01 vs. Sham group, # *P*<0.05 vs. Model group, ## *P*<0.01 vs. Model group.

Compared with the Sham group, the rate of weight gain in other groups decreased. However, when ANRIL expression was down-regulated, or miR-181b expression was up-regulated, the weight gain rate of mice was restored to a certain extent compared with the Model group. On D0, there was no significant difference in urea nitrogen and serum creatinine of each group, but urea nitrogen and serum creatinine levels were significantly increased in other groups compared with the Sham group on D48 (*P* < 0.001) (Fig 2A). However, when ANRIL expression was down-regulated, or miR-181b expression was up-regulated, urea nitrogen and serum creatinine levels in mice were significantly lower than those in Model mice (*P* < 0.05) (Fig 2B and 2C). The results of HE and PAS staining showed that, compared with the Sham group, other groups’ kidney and myocardial tissue were damaged to various degrees. However, renal and myocardial tissue damages in mice were less than that in the Model group, with higher ANRIL expression or lower miR-181b expression. TUNEL staining results showed that compared with the Model group, the apoptotic cells in renal and myocardial tissues increased in all the other groups. However, the number of apoptotic cells in the renal and myocardial tissues of mice was decreased compared with the Model group, with higher ANRIL expression or lower miR-181b expression (Fig 2D). The expressions of ANRIL and miR-181b in each group are shown in Fig 3A.

### ANRIL down-regulation or miR-181b up-regulation can regulate ATG5 expression and autophagy-related proteins in mice myocardium

Compared with the Sham group, ATG5 expression was significantly up-regulated in the Model, Model + sh NC, and Model+ NC agomir groups (*P* < 0.01). Compared with the Model group, ATG5 expression was significantly down-regulated in the Model+sh lncRNA ANRIL and Model+miR-181b agomir groups (*P* < 0.01) (Fig 3A). Compared with the Sham group, the expressions of ATG5, Beclin1, and LC3II/I in the Model, Model + sh NC, and Model+ NC agomir groups were significantly up-regulated. In contrast, p62 expression was significantly down-regulated (*P* < 0.01). Compared with the Model group, the expressions of ATG5, Beclin1, and LC3II/I protein were significantly down-regulated in Model + sh lncRNA-ANRIL and Model+miR-181b agomir groups, while p62 expression was significantly up-regulated (*P* < 0.01) (Fig 3B, S1 Fig).

### Expressions of ANRIL and miR-181b in CMECs treated with Indoxyl-Sulfate (IS)

**Fig 4:**
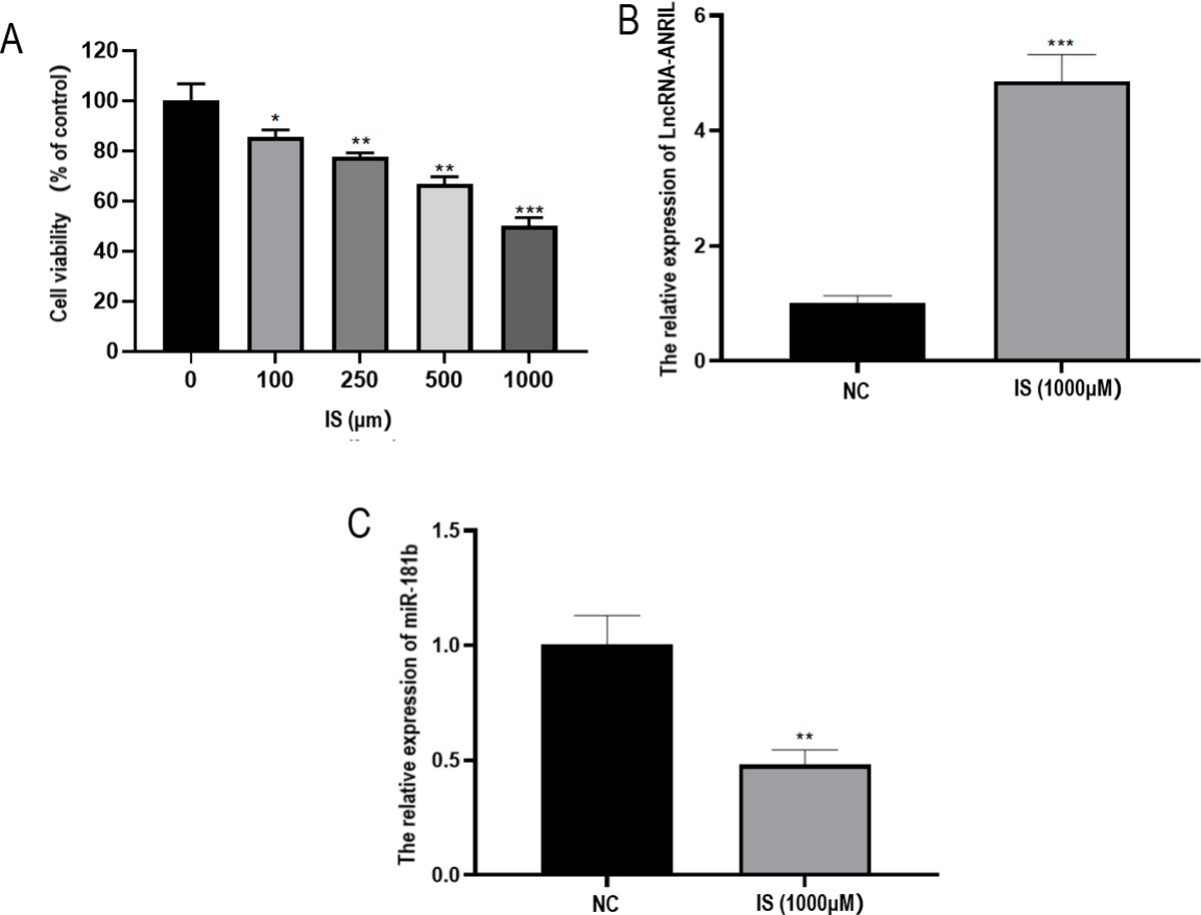
Effect of IS on the expression of lncRNA-ANRIL and miR-181b in CMECs. (A) Effects of 0, 100, 250, 500, and 1000 μmol/L IS on the proliferation of CMECs detected by CCK-8. (B) lncRNA-ANRIL expression detected by qRT-PCR. (C) miR-181b expression detected by qRT-PCR. *P < 0.05.

CMECs treated with IS can simulate the cell model of uremic cardiomyopathy in vitro. In this study, CMECs were treated with complete medium containing 0, 100, 250, 500, and 1000 μmol/L IS for 48h, and CCK-8 experiment was performed to detect the activity of cells. The results showed that 1000 μmol/L IS was the best drug concentration (Fig 4A). Compared with the NC group, ANRIL expression in CMECs significantly increased (*P* < 0.001) (Fig 4B), and the miR-181b expression significantly decreased when the concentration of IS was 1000 mol/L (*P* < 0.01) (Fig 4C).

### Extraction and identification of exosomes from CMECs

**Fig 5:**
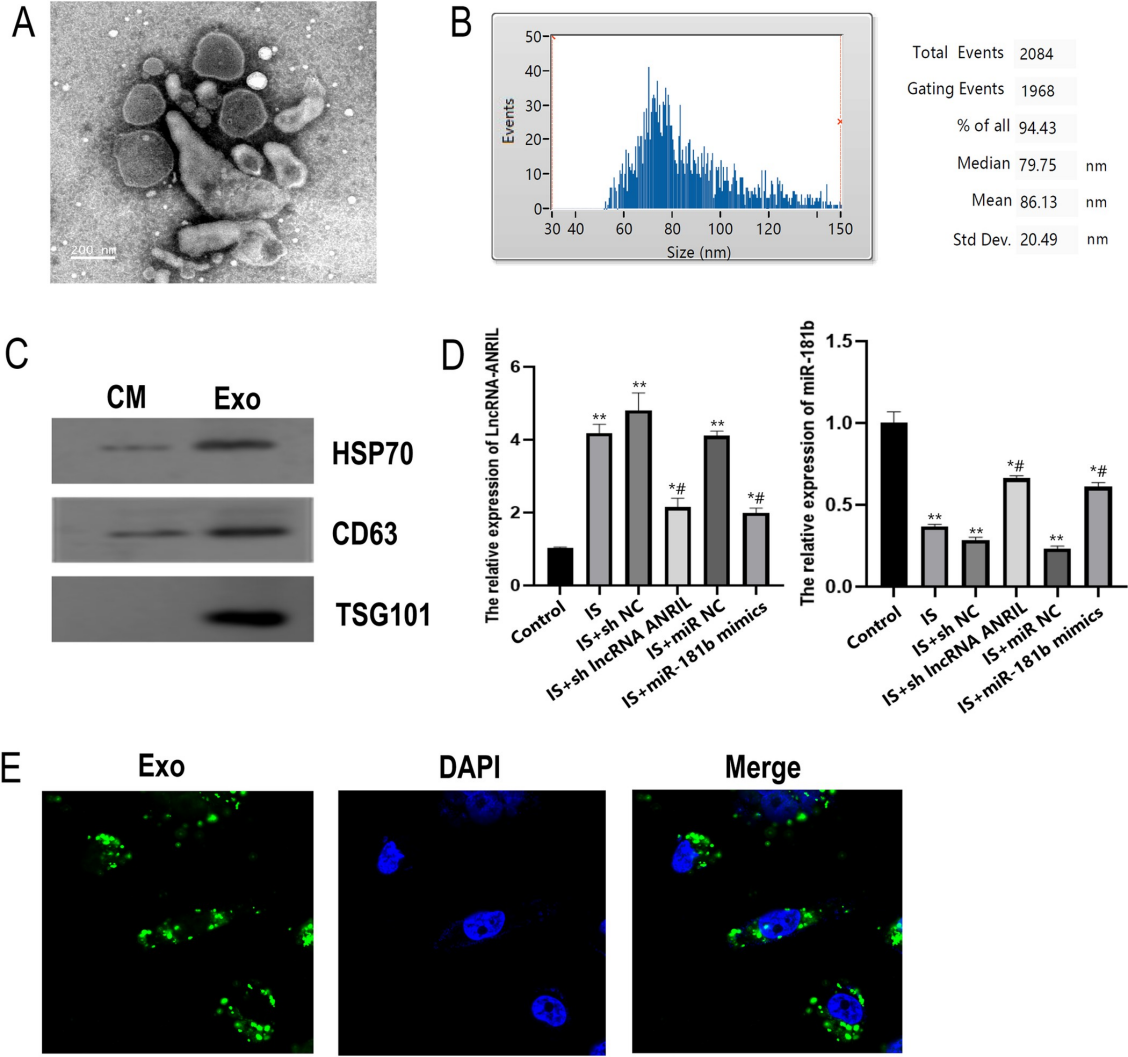
Identification of CMECs derived exosomes and its transfer in CMs. (A) Electron micrograph of exosomes extracted by ultracentrifugation (bar = 200nm). (B) Nano tracking technique (NTA) to detect the diameter of exosomes. (C) Expressions of HSP70, CD63, and TSG101 in exosomes. (D) Expression of lncRNA-ANRIL and miR-181b detected by qRT-PCR. (E) Laser confocal detection of exosomes transfer. * *P*<0.05 vs. Control group, ** *P*<0.01 vs. Control group, # *P*<0.05 vs. IS group.

The transmission electron microscopy of exosomes is shown in Fig 5A, which shows that round particles with a diameter slightly less than 200nm can be found in the figure, indicating that the exosomes are successfully separated and extracted. The results of particle size detection are shown in Fig 5B, which shows that the particle size of most extracted particles was about 80nm, conforming to the expected exosome characteristics. The expressions of HSP70, CD63, and TSG101 are shown in Fig 5C and S2 Fig. Compared with the control group, ANRIL expression in exosomes of IS, IS+sh NC and IS+miR NC groups significantly increased (*P* < 0.01). Compared with IS group, ANRIL expression in exosomes of IS+sh lncRNA ANRIL and IS+miR-181b mimics groups significantly decreased (*P* < 0.05). However, the expression trend of miR-181b was opposite to that of ANRIL (Fig 5D), which were enriched in exosomes but not in cells without exosomes. The exosomes from CMECs were labeled with green fluorescence, and the nuclei of CMs with blue. The uptake of exosomes by CMs was detected by fluorescence confocal assay., which showed that the exosomes (green fluorescence) were clearly visible in CMs (blue fluorescence), indicating that exosomes from CMECs can transferred into CMs (Fig 5E).

### ANRIL down-regulation or miR-181b up-regulation can regulate the expressions of ANRIL, ATG5, miR-181b, and autophagy-related proteins in CMs

**Fig 6:**
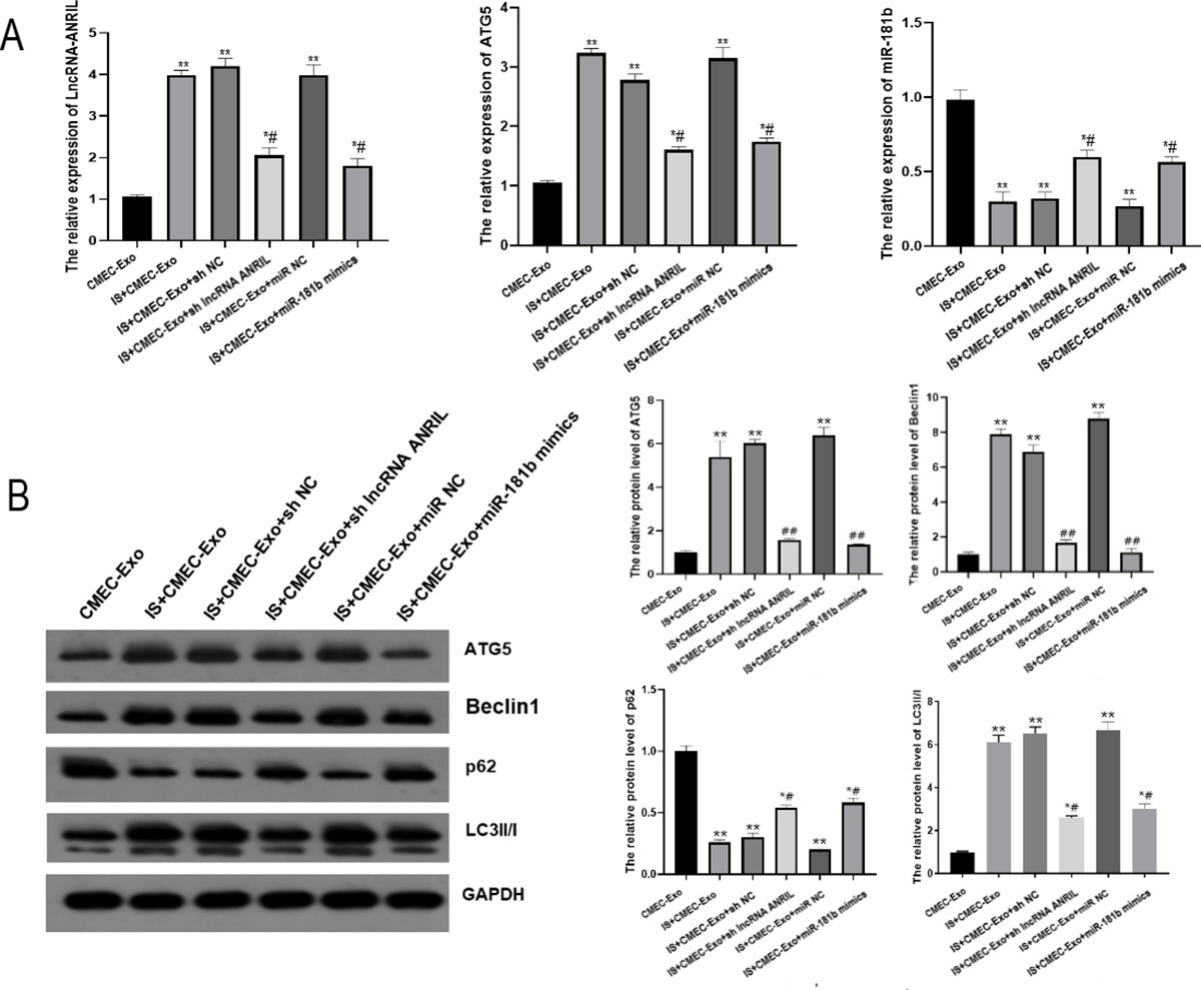
Effects of lncRNA-ANRIL down-regulation or miR-181b up-regulation on the expressions of lncRNA-ANRIL, ATG5, miR-181b, and autophagy-related proteins in CMs. (A) Expressions of lncRNA-ANRIL, ATG5, and miR-181b detected by qRT-PCR. (B) Protein expressions of ATG5, Beclin1, p62, and LC3 detected by Western blot. * *P*<0.05 vs. Control group, ** *P*<0.01 vs. Control group, # *P*<0.05 vs. IS group, ## *P*<0.01 vs. IS group.

Compared with the CMEC-Exo group, the expressions of ANRIL and ATG5 were significantly up-regulated, while miR-181b expression was significantly down-regulated in other groups (*P* < 0.05). Compared with the IS+CMEC-Exo group, the expressions of ANRIL and ATG5 were significantly down-regulated, while miR-181b expression was significantly up-regulated in the IS+CMEC-Exo+sh lncRNA ANRIL and IS+CMEC-Exo+miR-181b mimics groups (*P* < 0.05) (Fig 6A). Compared with the CMEC-Exo group, the expressions of ATG5, Beclin1, and LC3II/I in IS+CMEC-Exo p, IS+CMEC-Exo+sh NC, and IS+CMEC-Exo+miR NC groups were significantly up-regulated, while p62 expression was significantly down-regulated (*P* < 0.05). Compared with the IS+CMEC-Exo group, the expression of ATG5, Beclin1 and LC3II/I were significantly down-regulated in IS+CMEC-Exo+sh lncRNA ANRIL and IS+CMEC-Exo+miR-181b mimics groups, while the p62 expression was up-regulated considerably (*P* < 0.05) (Fig 6B, S3 Fig).

### Results of double fluorescein reporter gene experiment

**Fig 7:**
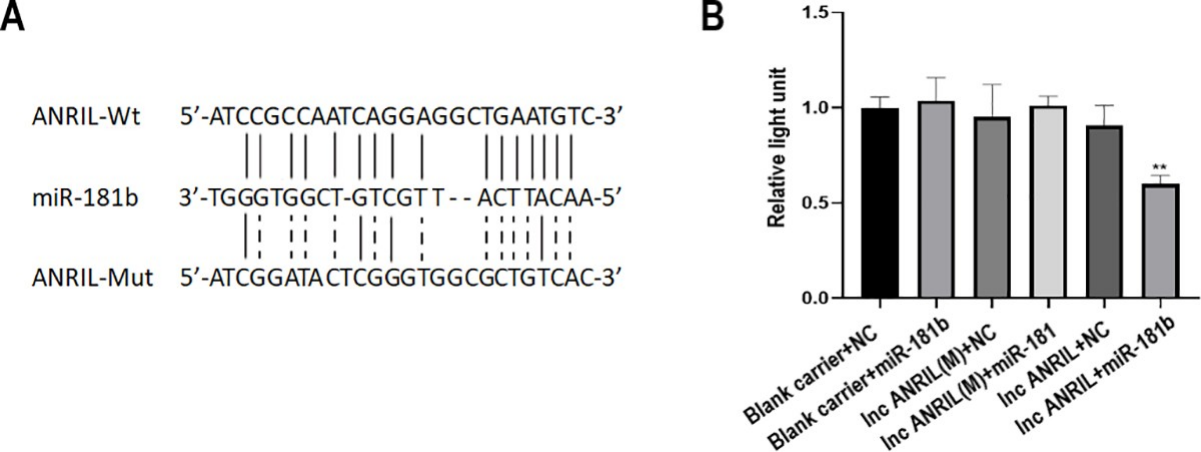
ANRIL was a target of miR-181b. (A) Complementary sequences of miR-181b contained by 3′UTR of ANRIL. (B) Interaction between ANTIL and miR-181b validated by Luciferase reporter gene assay. *P < 0.05.

In this study, the binding of ANRIL with miR-181b was detected with double luciferase assay, showing that the lncRNA-ANRIL + miR-181b group’s fluorescence intensity significantly decreased (Fig 7A and 7B), indicating that ANRIL can bind to miR-181b.

## Discussion

CVD in patients with uremia is a significant clinical problem, as the leading cause of death in uremia, which is mainly uremic cardiomyopathy and ischemic heart disease in uremia, With the heart’s pathological changes including left ventricular hypertrophy, myocardial interstitial fibrosis, and microvascular disease, accompanied by the loss of myocardial cells. Autophagy is a highly conserved intracellular protein recycling mechanism, leading to programmed cell death distinct from apoptosis and independent of the caspase pathway. And myocardial autophagy usually occurs in a variety of CVD. Studies have shown that CMs are particularly sensitive to changes in intracellular environment induced by autophagy. Inhibition of autophagy can improve cardiac function in patients with dilated cardiomyopathy [11, 12]. However, there are few studies on the mechanism of autophagy in uremic CVD.

lncRNAs, a class of non-coding RNAs with a length greater than 200 nt, are essential regulators of various cellular processes, which are important in biological function, with expression changes closely related to the occurrence and development of various diseases [13, 14]. It is reported that several lncRNAs are involved in the regulation of autophagy, but few studies have confirmed their regulatory role in cardiac autophagy. ANRIL, a long non-coding RNA 3.8kb long, is an essential genetic sensitive site of CHD [15]. Compared with healthy people, ANRIL expression in peripheral blood of patients with CHD significantly increased. In this study, the uremic model was established with 5/6 nephrectomy, which showed that compared with the Sham group, ANRIL expression in the Model group’s myocardial tissue increased.

ATG5 is an expression product of autophagy-relatedautophagy-related genes involved in forming double-membrane structure of autophagy precursor [16]. Beclin-1 is a important autophagy promoting gene in mammals acting on autophagy nucleation as a key protein to start autophagy [17]. LC3 is encoded by yeast autophagy-related gene 8 (ATG8) in mammalian cells belonging to a family of proteins that can mediate the cell membrane’s transport and binding [18]. The expressions of these three proteins were positively correlated with autophagy activity. P62 is a phosphorylated protein encoded by proto-oncogene c-myc, which was synthesized in the cytoplasm, expressing in nucleus [19]. When autophagy occurs, p62 is continuously degraded in the cytoplasm, with decreasing expression; when autophagy is blocked, p62 accumulates in the cytoplasm, with increasing expression. In this study, we found that compared with the sham group, the expressions of ATG5, beclin-1 and LC3 increased, and p62 expression decreased in the model group, suggesting that uremia can increase autophagy in mouse myocardium, which is consistent with Gong et al. [20], but reversed when ANRIL expression was knocked down in mice with uremia.

miR-181b is a member of miR-181 family. In recent years, miR-181b has been a hot topic in CVD. Guo et al. found that miR-181b expression in plasma of patients with CHD was significantly lower than that of healthy people [21]. CU copier et al. showed that miR-181b was down regulated in myocardial tissues of DCM mice [22]. In this study, compared with the Sham group, miR-181b expression in the myocardium of mice with uremia was significantly down-regulated. However, when ANRIL expression was knocked down in mice with uremia, this result was reversed, with a regulatory site between ANRIL and miR-181b, indicating the regulatory role of ANRIL in CVD realized through the regulation of miR-181b.

Exosomes are extracellular vesicles mediating intercellular signal communication by delivering mRNA, miRNAs, lncRNAs, proteins, and other active substances to receptor cells [23]. Vascular endothelial cells are important effector cells of angiogenesis. In recent years, researchers have found that exosomes derived from vascular endothelial cells play an important role in CVD, tumors, and sepsis [24, 25]. According to Huang et al., human umbilical vein endothelial cells (HUVEC) treated with ox LDL could promote the polarization of M2 macrophages by releasing exosomes rich in metastasis-associated lung adenocarcinoma transcript 1 (MALAT1) [26]. Halkein et al. also found that endothelial cells could release miR-146a rich exosomes under the action of 16-kDa N-terminal prolactin fragment (16K PRL) to act on CMs and reduce myocardial metabolism [27]. In this study, CMECs could release the exosomes rich in ANRIL under IS and promote autophagy in CMs. When ANRIL expression in CMECs was knocked down, the autophagy of CMs was alleviated.

## Conclusion

In conclusion, this study demonstrated that CMECs can regulate the autophagy of CMs by releasing exosomes containing ANRIL and miR-181b, with a negative regulatory relationship between ANRIL and miR-181b. This result provides a novel and promising alternative approach for the future treatment of uremic CVD. Nevertheless, this study carried out in vivo and in vitro experiments on ANRIL/miR-181b, yet the relevant results have not been verified by clinical data, requiring further research.

## Supporting information

S1 Fig As full as possible length gels and blots for Fig 3B. (TIF)

S2 Fig As full as possible length gels and blots for Fig 5C. (TIF)

S3 Fig As full as possible length gels and blots for Fig 6B. (TIF)
