# Supplementary figures and images for: Mechanism of lncRNA-ANRIL/miR-181b in autophagy of cardiomyocytes in mice with uremia by targeting ATG5
Source: PLoS One. 2021 Sep 1;16(9):e0256734. doi: 10.1371/journal.pone.0256734 (PMC8410126; doi:10.1371/journal.pone.0256734)

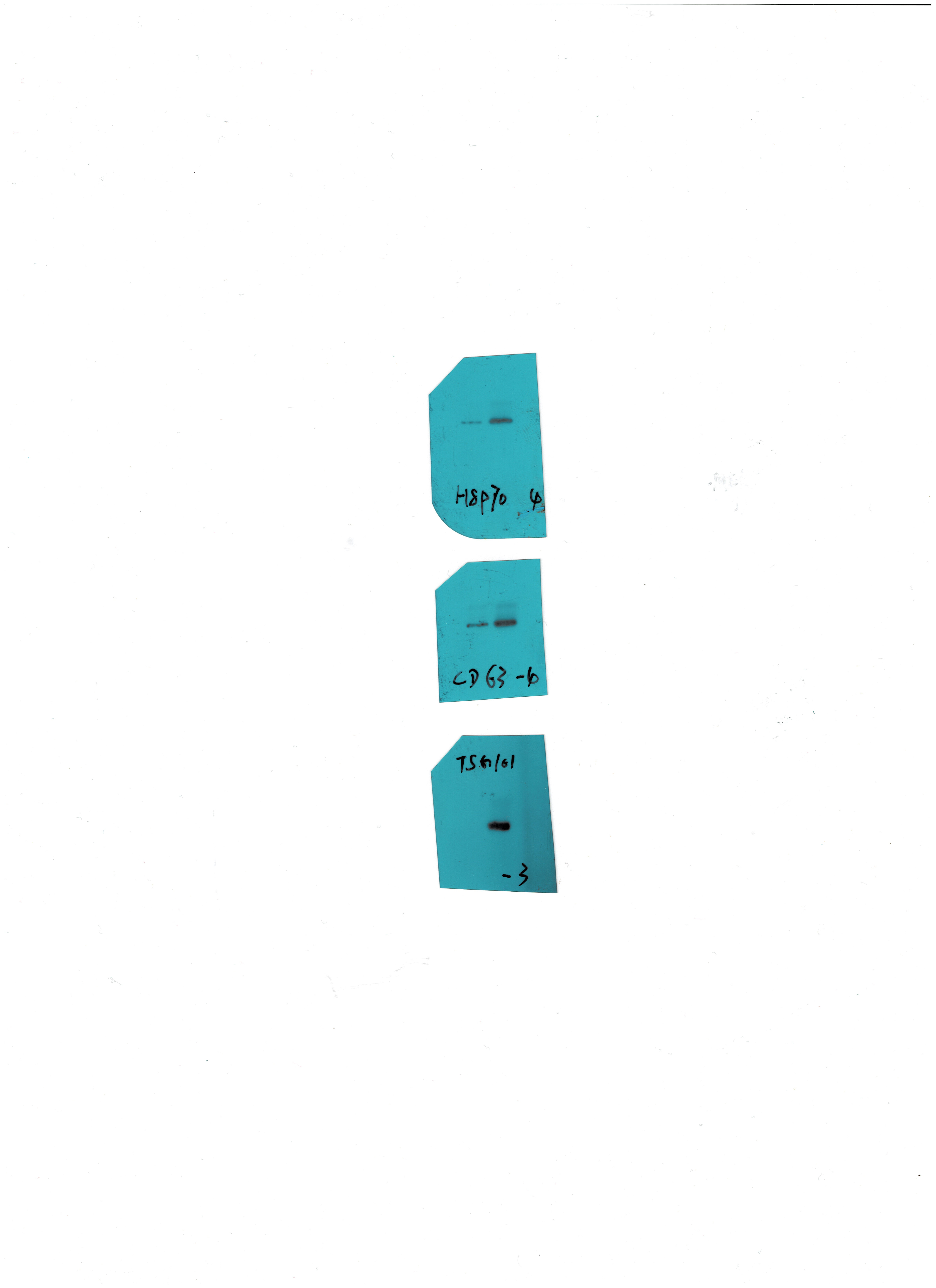

Supplement: S2 Fig — (TIF) [file pone.0256734.s002.tif]
